# Improving the Thermal Stability and Flame Retardancy of Epoxy Resins by Lamellar Cobalt Potassium Pyrophosphate

**DOI:** 10.3390/polym14224927

**Published:** 2022-11-15

**Authors:** Qinghong Kong, Lan Li, Manman Zhang, Huiyu Chai, Weixi Li, Fang Zhu, Junhao Zhang

**Affiliations:** 1 School of Emergency Management, Jiangsu University, Zhenjiang 212013, China; 2 School of Environmental and Chemical Engineering, Jiangsu University of Science and Technology, Zhenjiang 212013, China

**Keywords:** lamellar cobalt potassium pyrophosphate, thermal stability, smoke suppression, flame retardancy, physical barrier, catalytic carbonization

## Abstract

In order to improve the fire retardancy of epoxy resin (EP), lamellar cobalt potassium pyrophosphate (LCPP) nanocrystal whiskers with a length of 100–300 nm were designed and synthesized by a liquid technique. LCPP with high thermal stability was blended into EP to prepare the EP/LCPP composites. The results show that the EP/LCPP composites have higher thermal stability and produce more residues compared to pure EP. The combustion results display that the LOI value of the EP/10wt%LCPP composites was significantly improved to 35.9%, and the EP/6wt%LCPP composite can reach a UL-94 V-1 rating. Additionally, the peak heat release rate and peak smoke production rate of the EP/10wt%LCPP composites dramatically decreased by 43.8% and 48.5%, respectively. The improved flame retardancy and smoke suppression are mainly attributed to the inherent physical barrier of LCPP and the excellent catalytic carbonization ability of LCPP.

## 1. Introduction

Epoxy resin (EP) has excellent mechanical, insulation, and bonding properties. It is diffusely applied in electronics, transportation, construction, national defense, machinery, aerospace, and other fields [1,2,3]. However, flammability is one of the biggest shortcomings of epoxy resin, with an oxygen index of only about 19%, which can easily lead to fire [4,5]. Therefore, it is necessary to modify epoxy resin to have better flame retardancy and excellent thermodynamic properties.

Halogen-containing compounds are currently the most common flame retardants, which have high flame retardancy and good mechanical properties [6,7,8]. Nevertheless, once halogen-containing flame-retardant polymer composites are burned, large amounts of corrosive and toxic gases are released, which easily result in environmental pollution, causing secondary damage. Halogen-free flame retardants have become a key technology in the fire retardant field because of their halogen-free, low toxicity, and good flame retardancy, such as layered double hydroxide (LDH), graphene oxide (GO), metal phosphate, and transition-metal-based compounds [9,10,11,12].

Among them, metal phosphate was employed as a flame retardant based on the nanosized, catalytic carbonization, and high-efficiency flame retardant effect of phosphorus-containing flame retardants [13,14,15]. For example, α-zirconium phosphate was modified by intumescent flame retardant and blended with polylactic acid. The results indicated that adding modified α-zirconium phosphate remarkably improved the fire retardance of polylactic acid composites, but their thermal stability ultimately decreased [16]. In order to ameliorate the thermal stability of α-zirconium phosphate-based polymer composites, octa-epoxy polyhedral oligomeric silsesquioxane (POSS) as a synergist was selected for improving the thermal stability of EP nanocomposites containing layered zirconium phenylphosphate (ZrPP) due to the high decomposition temperature of POSS [17]. By blending nanoporous nickel phosphate VSB-1, the ammonium polyphosphate and pentaerythritol (petol) matrix made polypropylene composites pass UL-94 V-0 rating, with a limiting oxygen index (LOI) value of as high as 35.5% [18]. Based on this, metal phenylphosphonate was recently designed and synthesized for application in polymers due to suitable thermal stability [19,20]. The TGA result indicated that ferric pyrophosphate (FePP) with IFR remarkable boosted the thermostability and fire retardance of the PP/IFR/FePP composites [21]. Consequently, designing an efficient flame-retardant phosphoric acid-based transition metal compound is essential for improving the flame-retardant properties of polymers.

In this work, new lamellar cobalt potassium pyrophosphate (LCPP) was successfully synthesized through a mixed solvothermal technique. LCPP with a layered structure is conducive to its dispersion in a polymer matrix, while the high thermal stability can improve the thermal stability, flame retardancy, and smoke suppression of EP composites. EP composites with only 6wt%LCPP passed a UL-94 V-1 rating, and the LOI value of the EP composites with 10wt%LCPP significantly improved from 25.8% to 35.9% of pure epoxy resin. The peak heat release rate (HRR), total heat release (THR), peak smoke production rate (PSPR), and total smoke production (TSP) of the EP/10wt%LCPP composites dramatically decreased by 43.8%, 34.3%, 48.5%, and 27.4%, which are mainly due to the physical barrier and excellent catalytic carbonization of LCPP.

## 2. Experimental Section

### 2.1. Materials

Cobalt acetate (ACS, ≥99.8%), potassium chloride (ACS, ≥99.8%), potassium pyrophosphate (ACS, ≥99.8%), diaminodiphenylmethane (DDM, about 75% of 4,4′-diamino diphenylmethane, about 25% of polyphenyl polyaminomethane) and acetone were obtained from Sinopharm Chemical Reagent Co., Ltd. (Shanghai, China). Epoxy resin (NPEL128, epoxy equivalent per weight with 180–190 g/eq, viscosity from 8000 to 9000 mPa·s at 25 °C, density about 1.16 g/cm^3^ at 25 °C.) was purchased from South Asia Electronic Materials Co., Ltd. (Kunshan, China).

### 2.2. Synthesis of Cobalt Potassium Pyrophosphate

Cobalt potassium pyrophosphate was synthesized by a liquid technique. A total of 0.498 g of Co(CH_3_CO_2_)_2_, 0.768 g of K_4_P_2_O_7_, and 7.460 g of KCl was dissolved in 30 mL deionized H_2_O with magnetic stirring for 30 min at 25 °C, which was poured into a 50 mL teflon-lined autoclave, and heated at 120 °C for 4 h. The products were washed four times with ethanol and water, and then oven-dried at 60 °C for 12 h.

### 2.3. Preparation of EP/LCPP Composites

A certain amount of LCPP was blended with epoxy resin to prepare the EP/LCPP composites. To obtain EP/LCPP composites with satisfactory dispersion, LCPP and epoxy resin were mixed into acetone with ultrasonic dispersion for 20 min. After that, vacuum drying was used to remove the solvent at 85 °C, and DDM was added into the mixture at 90 °C by agitating vigorously for 15 min. The mixture was put into a vacuum dryer at 100 °C for 5 min to expel bubbles, and fleetly poured into the preheated silicone rubber mold. Subsequently, the mixture was heated at 110, 130, and 150 °C for 2 h, respectively. The resulted products were demolded and named as the EP/LCPP composites. The ingredients of the EP/LCPP composites were presented in Table 1.

### 2.4. Characterization

The X-ray powder diffraction (XRD) pattern of LCPP was obtained from a MAX-RB X-ray diffractometer (Rigaku Co., Tokyo, Japan), working at 40 kV and 100 mA and assembled with graphite-Cu Kα radiation (λ = 1.5418 Å). The scanning electron microscopy (SEM) imaging of LCPP and the residues of the EP/LCPP composites after CCT were gained through a scanning electron microscope (Zeiss, EVO MA15, Jena, Germany). The distribution of the elements in LCPP was investigated using energy dispersive X-ray spectroscopy (EDS, FEI Company, Hillsboro, OR, USA). Transmission electron microscopy (TEM, JEOL JEM-100SX) images of LCPP were characterized at 100 kV. Thermogravimetric (TG) curves of the EP/LCPP composites were obtained by a thermo-analyzer instrument (Q50, TA Instruments, New Castle, DE, USA), going up 10 °C/min in an N_2_ atmosphere. According to ASTM D 3801, the UL-94 ratings were tested on a burning chamber (Fire Testing Technology, Grinstead, UK) with the sizes of 127 × 12.7 × 3.2 mm^3^. LOI values were obtained with dimensions of 130 × 6.5 × 3.2 mm^3^, using an oxygen index model instrument (HC-2, Nanjing, China) based on ASTM D 2863. The combustion data of the EP/LCPP composites were acquired from a cone calorimeter (FTT, Grinstead, UK), according to ISO 5660-1, and each specimen (100 × 100 × 4 mm^3^) was irradiated at a heat flux of 50 kW/m^2^.

## 3. Results and Discussion

### 3.1. Structure and Morphology of Cobalt Potassium Pyrophosphate

**Figure 1:**
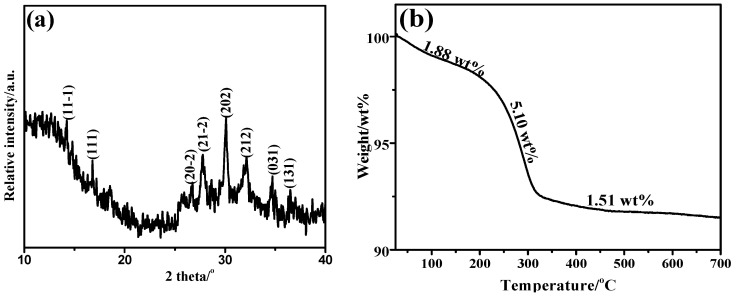
(**a**) XRD pattern of LCPP; (**b**) TGA curve of LCPP.

Figure 1a is the XRD pattern of the product, and all diffraction peaks are assigned to cobalt potassium pyrophosphate (JCPDS No. 80-1172). No peaks were detected from other phosphites or phosphates, indicating the product is pure cobalt potassium pyrophosphate. To further characterize the structure of LCPP, Figure 1b exhibits the TGA curve of LCPP for investigating the thermal degradation behavior of LCPP. The thermal decomposition of LCPP occurs in three stages. The mass loss before 200 °C is only about 1.88 wt%, which is mainly from some absorption water in the LCPP. The mass loss of the second stage was 5.10 wt% between 200 and 320 °C, which we assign to the pyrophosphate translating into metaphosphoric acid, while the third stage (from 320 to 650 °C) is ascribed to the decomposition of metaphosphoric acid. The phosphate groups were converted to phosphorous after 650 °C. According to the above characterization, we can conclude that LCPP nanocrystal whiskers with higher thermal stability were successfully prepared.

**Figure 2:**
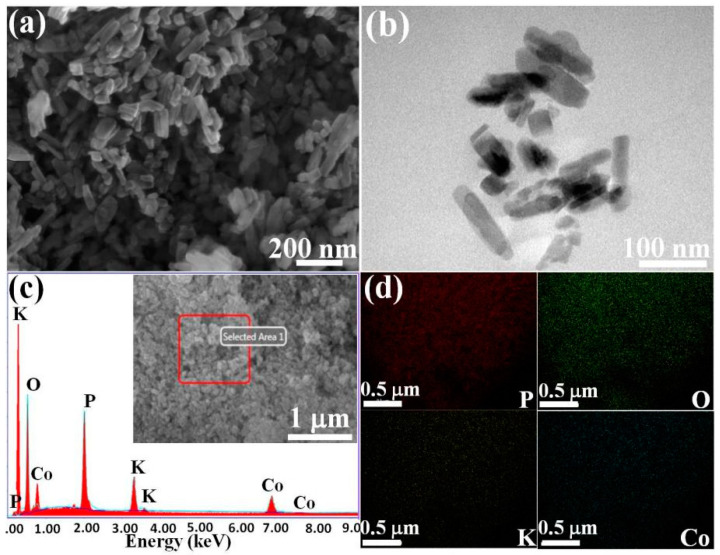
(**a**) SEM image of LCPP; (**b**) TEM image of LCPP; (**c**) EDS spectrum of LCPP, and the inset is the corresponding SEM image; (**d**) SEM elemental mapping images of LCPP nanocrystal whiskers from the area in the inset of (**c**).

The SEM image in Figure 2a shows that the shape of LCPP is nanowhisker, with a length of 100–300 nm, and the yield is more than 95%. In order to clearly apperceive the microstructure of LCPP, TEM images are also provided in Figure 2b, which is consistent with the result of the SEM images. The EDS and mapping images of the LCPP nanocrystal whiskers are shown in Figure 2c,d. The EDS spectrum indicates that LCPP consists of K, Co, P, and O elements, and the inset of Figure 2c provides the SEM image used for testing the elemental mapping images of LCPP. The elemental mapping images also prove that LCPP consists of K, Co, P, and O elements, which are uniformly distributed in the LCPP whiskers [22,23].

### 3.2. Thermal Stability of EP/LCPP Composites

**Figure 3:**
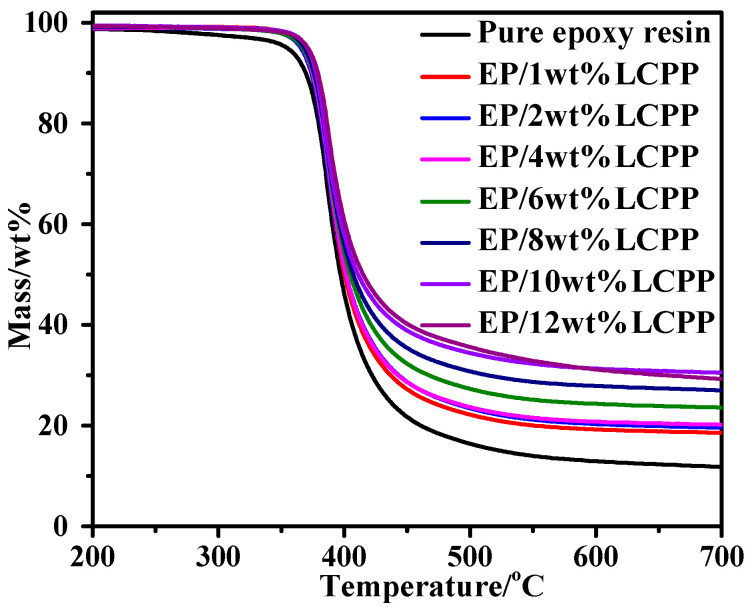
TGA curves of pure epoxy resin and the EP/LCPP composites.

In order to investigate the effects of LCPP on the EP/LCPP composites, the TGA was measured, as shown in Figure 3, and the relevant data are provided in Table 2. The results indicate that the T_5%_ (the temperature at 5% mass loss) of pure EP is 354 °C, indicating that the main pyrolysis stage is ascribed to the pyrolyzation of the epoxy resin networks. However, the T_5%_ of the EP/LCPP composites increased to 366 °C when the content of LCPP is only 1 wt%. When the content of LCPP increased from 1 wt% to 10 wt%, the thermal stability of the EP/LCPP composites ameliorated, exhibiting the observable elevation in T_5%_ and T_50%_ (the temperature at 50% mass loss), which we assign to the higher initial thermolysis temperature of LCPP. However, when increasing the content of LCPP to 12 wt%, the thermal stability of the EP/LCPP composites began to decrease due to poor dispersion. Moreover, the char residue of the pure EP is about 11.8 wt%, whereas those EP/LCPP composites with 1, 2, 4, 6, 8, and 10wt%LCPP have 18.6, 19.6, 20.2, 23.6, 27.0 and 30.5 wt% residues at 700 °C, respectively, indicating that the introduction of LCPP into the EP matrix boosts the production of char because of the excellent catalytic charring ability of LCPP [24]. However, with the incorporation of 12wt%LCPP, the char residues slightly decreased to 29.3 wt% at 700 °C, which is ascribed to the uneven dispersion of LCPP in the epoxy resin. The increase in the residual yield is an important index for evaluating thermostability at high temperatures, [25,26] meaning the incorporation of LCPP can evidently improve the thermostability of the EP/LCPP composites.

### 3.3. Combustion Performance of EP/LCPP Composites

The fire retardancy of the EP/LCPP composites was evaluated via the LOI values and the UL-94 measures, as shown in Table 1. Pure EP exhibits no UL-94 level, and the LOI value is 25.8%. However, the LOI values of the EP/LCPP composites increased when the content of LCPP was increased. When the LCPP content reached 10 wt%, the LOI value was as high as 35.9%. In addition, when increasing the content of LCPP, the (t_1_ + t_2_) values in the UL-94 testing decrease gradually, and this can pass V-1 when the LCPP content is 6 wt%. When the content of the LCPP rose to 10 wt%, the (t_1_ + t_2_) value of the EP/LCPP composite was only 21.2 s. Nevertheless, when the amount of LCPP further increases to 12 wt%, the LOI value of the EP/LCPP composites slightly decreases because of the uneven dispersion of LCPP in the EP matrix. The (t_1_ + t_2_) values of the UL-94 measures has the same trend as those of the LOI. According to the above results, 10wt%LCPP is the best content with which to prepare the EP/LCPP composites for excellent fire retardance. The superior fire retardance is basically ascribed to the physical barrier effect, the good synergistic fire retardance of P-containing species, and the catalytic charring of LCPP [27,28].

**Figure 4:**
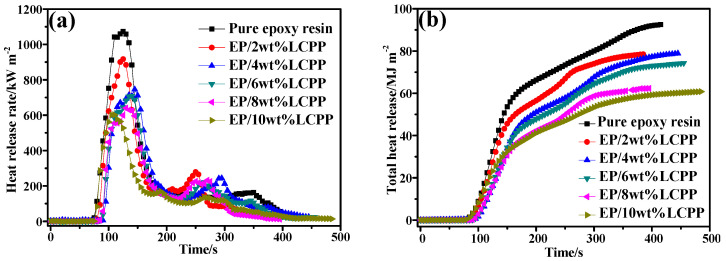
Heat release of pure EP and the EP/LCPP composites; (**a**) HRR profiles; (**b**) THR profiles.

**Figure 5:**
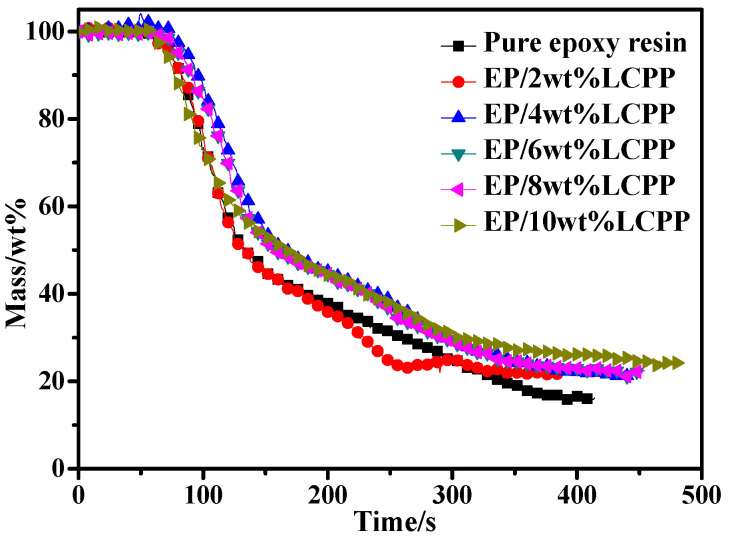
Mass loss curves of EP and the EP/LCPP composites.

**Figure 6:**
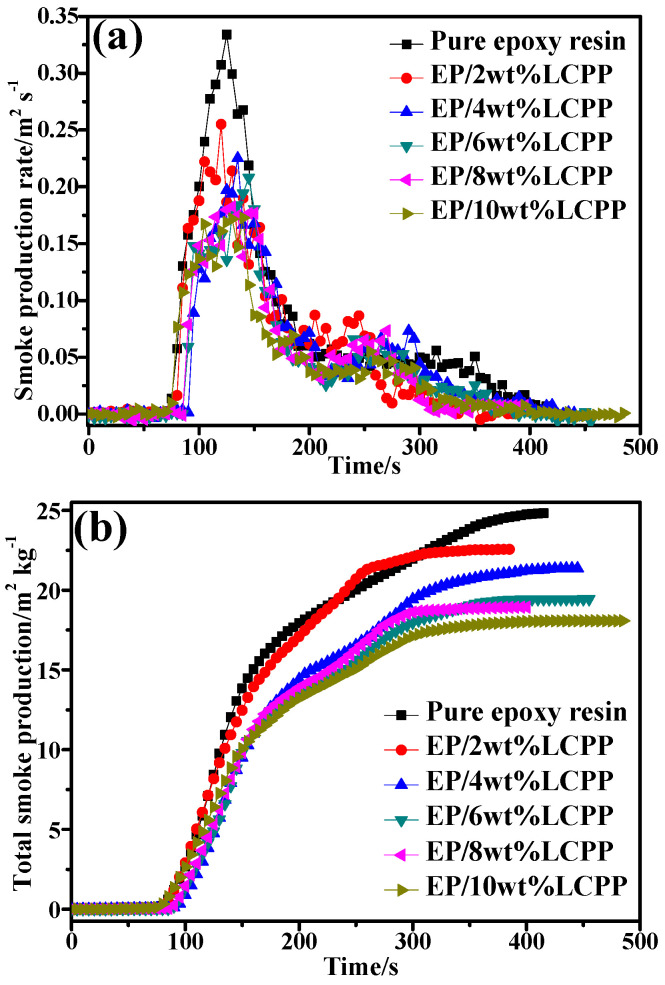
Smoke gas release of EP and the EP/LCPP composites; (**a**) SPR profiles; (**b**) TSP profiles.

For the purpose of understanding the effect of LCPP on improving the fire retardancy of the EP/LCPP composites, the combustion of small specimens was simulated by cone calorimeter tests. The important indicators, such as time to ignition (TTI) HRR, THR, fire growth index (FGI), fire performance index (FPI), SPR, and TSP are shown in Table 3 and Figure 4, Figure 5 and Figure 6. As shown in Table 3, the pure EP had a TTI value of 77 s, and the TTI values of EP/2wt%LCPP and EP/4wt%LCPP increased to 83 s and 120 s, which demonstrated that the incorporation of LCPP can inhibit the decomposition and combustion of EP due to the high thermal stability of LCPP, agreeing well with the TG results aforementioned. When the amount of LCPP added increased to 10 wt%, the TTI value decreased to 83 s; this is still higher than that of pure EP. The HRR profiles of pure EP and the EP/LCPP composites are shown in Figure 4a. Once pure EP is ignited, it will burn violently, and the peak value of HRR is as high as 1073.0 kW/m^2^. In comparison to pure EP, the PHRR value of the EP/LCPP composites with 2wt%LCPP declined to 917.2 kW/m^2^. When adding 4, 6, 8, and 10wt%LCPP, the PHRR values greatly decreased to 917.2, 745.8, 707.8, 640.0, and 602.6 kW/m^2^, a reduction of about 30.5%, 34.0%, 40.4%, and 43.8%, respectively. The results clearly show that incorporating LCPP can significantly enhance the fire retardance of EP composites. On the one hand, the laminated structure of LCPP has a physical barrier effect; on the other hand, the cobalt-containing group has good catalytic charring ability, boosting the carbon-based residues on the upper surface of the EP/LCPP composites, thereby inhibiting mass and heat transfer and reducing heat release [9,29].

Figure 4b shows the THR profiles of pure EP and the EP/LCPP composites. The THR curves show that pure EP quickly releases a lot of heat and the THR value is as high as 92.5 MJ/m^2^. Meanwhile, with the introduction of 2, 4, 6, 8, and 10wt%LCPP into the EP, the THR values distinctly dwindle to 78.5, 78.8, 74.2, 62.3, and 60.8 MJ/m^2^, which shows that more organic species from the pyrolysis of EP were carbonized, and the formation of the carbonaceous residual layer covered the surface of the polymer to prevent the exchange of oxygen and heat with the outside world [30].

In order to further elucidate the fire retardancy effect of LCPP on the EP composites in the condensed phase, the mass loss profiles are shown in Figure 5. Pure EP has the lowest residual amount: only 19.0 wt% at 350 s. With the incorporation of LCPP, the residues from the EP composites improved gradually, and the residue of the EP/10wt%LCPP composites reached 27.3 wt%. This increase in the residues is mainly ascribed to the superior catalytic and dehydration charring performance of LCPP. The results are consistent with the heat release trend [31,32]. In order to evaluate the flame retardancy more clearly, the flame retardancy index (FRI) values of pure EP and the EP/LCPP composites were calculated after cone calorimeter tests. The FRI was defined as the ratio of THR ∗ (pHRR/TTI) between the neat polymer and the corresponding thermoplastic composite containing only one flame. The results in Table 3 show that the FRI values increase gradually with increasing LCPP from 2 wt% to 10 wt%, and the FRI values are located in between 1 and 10, which indicates that the EP/LCPP composites exhibit good flame retardancy [33].

In fire disasters, smoke and toxic gases cause the greatest damage to life, followed by flame and heat. More than 70% of misfortune or death are caused by smoke and toxic gases in fire accidents [34]. Consequently, it is indispensable to investigate smoke and toxic gas emissions. The SPR profiles of pure epoxy resin and the EP/LCPP composites are presented in Figure 6a. When 2 wt% and 4wt%LCPP are incorporated into EP, the PSPR values of the EP/LCPP composites decrease to 0.25 and 0.22 m^2^/s from 0.33 m^2^/s of pure EP. With the addition of 6, 8, and 10wt%LCPP, the PSPR values of the EP/LCPP composites were drastically reduced to 0.20, 0.18, and 0.17 m^2^/s, respectively. This reduction in the PSPR value is propitious to fire rescue in fire disasters. Meanwhile, the TSP curves (as a function of time) for pure EP and the EP/LCPP composites are presented in Figure 6b, which are consistent with the PSPR curves. The TSP value of pure EP is 24.8 m^2^/kg. When the contents of LCPP are increased to 2, 4, 6, 8, and 10 wt%, the TSP values of the EP/LCPP composites decreased to 22.5, 21.3, 19.4, 18.9, and 18.0 m^2^/kg, respectively, being reduced by about 9.3%, 14.1%, 21.8%, 24.0%, and 27.4%, respectively, in comparison to pure epoxy resin; this is attributed to the production of a dense, carbon-containing protective layer between the flame zone and the EP/LCPP composites, thereby retarding the unburnt EP/LCPP composites from degrading into organic fragments and being converted into toxic gas [35,36].

**Figure 7:**
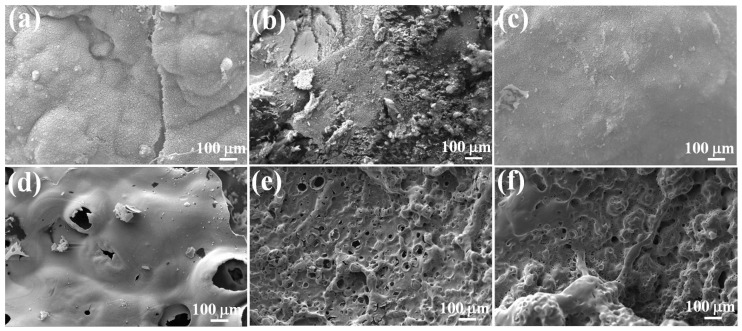
SEM images of the external char layers of (**a**) pure EP, (**b**) EP/2wt%LCPP, and (**c**) EP/10wt%LCPP; SEM images of internal char layers of (**d**) pure EP, (**e**) EP/2wt%LCPP, and (**f**) EP/10wt%LCPP.

In order to further explicate the mechanism of fire retardancy and smoke suppression for the EP/LCPP composites, the structures of the carbon-containing protective residues were examined through SEM characterization to obtain detailed information on decomposition and carbonization, as shown in Figure 7. The external char layer of pure EP is a consolidated plate-like carbon layer structure, and it is the fragile and incompact part in the internal char layer, which is due to the rapid decomposition, gasification, and combustion of the EP/LCPP composites. When the content of LCPP is enhanced, the external char layers of the EP/2wt%LCPP and EP/10wt%LCPP composites exhibited more fluffy and smooth carbon-containing residue layers, which is due to the superior catalytic charring ability of LCPP. Compared with pure EP, the internal char layer of the EP/2wt%LCPP composites is continuous and denser, and that of the EP/10 LCPP composites becomes thicker and denser due to the multicarbon remaining in the condensed phase, corresponding to the highly charred residues of the EP/10wt%LCPP composites. The above analysis proves that the carbon-containing protective layer formed on the surface of the EP/LCPP composites is firm and stable, which can effectively block the heat, mass, and oxygen transfer between the substrate material and combustion zone, boosting the fire retardancy and smoke suppression of the EP/LCPP composites [37,38].

## 4. Conclusions

In summary, LCPP nanocrystal whiskers were synthesized via a facile solvothermal method, blended into epoxy resin, and prepared as EP/LCPP composites. The characterizations showed that LCPP has a lamellar structure with a length of 100–300 nm, and the molecular formula is K_2_Co_3_(P_2_O_7_)_2_·2H_2_O. The TGA results revealed that the addition of LCPP ameliorated the thermostability of the EP/LCPP composites and observably enhanced the residual amount at 700 °C because of the superior catalytic charring ability of LCPP. The combustion results indicated that the LOI value of the EP/10wt%LCPP composite was significantly improved to 35.9%, and the EP/6wt%LCPP composite reached a UL-94 V-1 rating. Moreover, the PHRR and PSPR values of the EP/10wt%LCPP composites dramatically decreased by 43.8% and 48.5%, respectively. The excellent flame retardancy and smoke suppression capacity are ascribed to the inherent physical barrier of LCPP and the excellent catalytic performance of LCPP, which forms continuous and dense inorganic ceramic protection layers and efficiently prevents heat and mass transmission between the EP/LCPP composites and the flame zone.

## Figures and Tables

**Table 1:** Ingredients of the EP composites and the corresponding LOI values and UL-94 levels.

Samples	Components		Flame Retardancy		
	EP/wt%	LCPP/wt%	LOI/vol%	UL-94	
				(t_1_ + t_2_)/s	Rating
Pure epoxy resin	100	0	25.8	>50.0	NR
EP/1wt%LCPP	99	1	27.2	>50.0	NR
EP/2wt%LCPP	98	2	28.3	>50.0	NR
EP/4wt%LCPP	96	4	29.8	>50.0	NR
EP/6wt%LCPP	94	6	31.6	40.6	V-1
EP/8wt%LCPP	92	8	33.8	32.3	V-1
EP/10wt%LCPP	90	10	35.9	21.2	V-1
EP/12wt%LCPP	88	12	34.2	28.4	V-1

**Table 2:** The results of the TGA for pure epoxy resin and EP/LCPP composites.

Samples	T_5%_/°C	T_50%_/°C	Residues at 700 °C/wt%
Pure epoxy resin	354	397	11.8
EP/1wt%LCPP	366	400	18.6
EP/2wt%LCPP	369	404	19.6
EP/4wt%LCPP	369	404	20.2
EP/6wt%LCPP	368	404	23.6
EP/8wt%LCPP	370	411	27.0
EP/10wt%LCPP	371	415	30.5
EP/12wt%LCPP	366	410	29.3

**Table 3:** Combustion parameters obtained from the cone calorimeter test.

Samples	TTI/ s	Peak HRR/ kW m^−2^	THR/ MJ m^−2^	PSPR/ m^2^ s^−1^	TSP/ m^2^ kg^−1^	FRI	Residues/wt%
pure epoxy resin	77	1073.0	92.5	0.33	24.8	1.0	19.0
EP/2wt%LCPP	83	917.2	78.5	0.25	22.5	1.5	21.8
EP/4wt%LCPP	98	745.8	78.8	0.22	21.3	2.1	24.6
EP/6wt%LCPP	93	707.8	74.2	0.20	19.4	2.3	24.6
EP/8wt%LCPP	91	640.0	62.3	0.18	18.9	2.9	24.6
EP/10wt%LCPP	83	602.6	60.8	0.17	18.0	2.9	27.3

## Data Availability

Not applicable.
